# Professor Paulo Norberto Discher De Sá (1939-2022)

**DOI:** 10.1590/0004-282X-ANP-2022-M001

**Published:** 2022-08-08

**Authors:** Ylmar Correa, Hélio Afonso Ghizoni Teive

**Affiliations:** 1 Universidade Federal de Santa Catarina, Hospital Universitário, Departamento de Clínica Médica, Serviço de Neurologia, Florianópolis SC, Brazil.; 2 Universidade Federal do Paraná, Hospital de Clínicas, Departamento de Medicina Interna, Serviço de Neurologia, Curitiba PR, Brazil.

**Figure 1.**
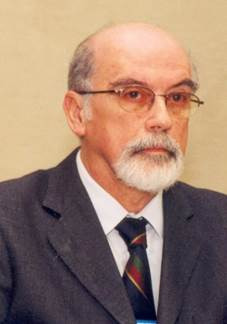
Professor Paulo Norberto Discher de Sá (1939-2022).

Professor Paulo Norberto Discher de Sá (Figure 1) was born in October 11th, 1939, in Lages, a small city in the highlands of Santa Catarina State, Brazil. When he was still young his family moved to the coastal city of Florianópolis, State’s capital. The interest in Medicine came from reading Ambroise Paré and William Osler biographies and Cronin’s Citadel in childhood. Professor Sá studied Medicine at the Federal University of Paraná, Curitiba, from 1958 to 1963, becoming a disciple of Professor Lysandro de Santos Lima, a well-known inspiring clinician. Between 1964 and 1965 he studied Neurology at the Guanabara State Public Servers Hospital, an important centre on Neurology training in Rio de Janeiro^1^ ^,^ ^2^ ^,^ ^3^. Back to Florianópolis Professor Sá worked at the Charity Hospital, whose Neurology service he would became chief. In 1968 he became the first neurologist to teach Neurology at Federal University of Santa Catarina. When the University Hospital was inaugurated in 1980, he assumed the Neurology service and later created the hospital Neurology Residence Program. Professor Sá also taught Neurology at UNISUL, a university in the nearby cities of Tubarão and Palhoça. Admired teacher, Professor Sá knew how to summarize complex matters on lectures and how to instigate the mystery of neurological examination on clinical rounds. Always cordial with his students, Professor Sá inspired the study of Neurology in many of them. Respected by his patients, he carefully paid visit daily to them on Charity and University Hospital, including on weekends and holidays. At the State Medical Ethics Council, he was known by his sharp and wise analysis. At the Brazilian Academy of Neurology, Professor Sá acted many years at the Teaching Commission and presided the Brazilian Congress of Neurology, at 2002 in Florianópolis. Professor Sá died on March 15th, 2022, after a long neurodegenerative disease, leaving his wife Regina, his son Daniel, neurologist established in the USA, his daughter Paula, architect, and grandchildren.
